# The outcomes of salvage robotic radical prostatectomy following radiation versus focal therapy: Does the primary treatment modality matter?

**DOI:** 10.1002/bco2.70019

**Published:** 2025-05-01

**Authors:** Alireza Ghoreifi, Lorenzo Storino Ramacciotti, Masatomo Kaneko, Luis G. Medina, Giovanni E. Cacciamani, Shiran Konganige, Manju Aron, Sarmad Sadeghi, Hossein Jadvar, Hooman Djaladat, Rene Sotelo, Mihir M. Desai, Inderbir S. Gill, Monish Aron, Andre Luis Abreu

**Affiliations:** ^1^ USC Institute of Urology, Catherine and Joseph Aresty Department of Urology, Keck School of Medicine University of Southern California Los Angeles California USA; ^2^ Center for Image‐Guided Surgery, Focal Therapy and Artificial Intelligence for Prostate Cancer, Keck School of Medicine University of Southern California Los Angeles California USA; ^3^ Department of Pathology Keck School of Medicine University of Southern California Los Angeles California USA; ^4^ Department of Medical Oncology University of Southern California Norris Comprehensive Cancer Center Los Angeles California USA; ^5^ Department of Radiology Keck School of Medicine University of Southern California Los Angeles California USA

**Keywords:** focal therapy, outcomes, prostatectomy, radiotherapy, robotics, salvage therapy

## Abstract

**Objectives:**

We aim to compare salvage robotic radical prostatectomy (sRRP) for recurrent prostate cancer (PCa) after primary radiation (RT) versus focal therapy (FT).

**Materials and Methods:**

Patients who underwent sRRP following primary local therapy for PCa were identified. Perioperative findings and functional/oncologic outcomes were compared in RT versus FT groups.

**Results:**

Overall, 112 patients were included, with 84 receiving RT and 28 FT as primary treatment. Median age and PSA were 68 years and 5.4 ng/mL, respectively. There was one rectal injury in the RT group. The overall 90‐day complications were significantly higher in RT group (33% vs. 11%, *p* = 0.03). On multivariable analysis, history of RT and prolonged operative time were associated with a higher rate of 90‐day complications. The 6‐ and 12‐month continence rates were higher in FT group (50% vs. 20%, *p* = 0.02 and 69% vs. 33%, *p* = 0.03). Potency at 12 months was better preserved in FT group (46% vs. 12%, *p* = 0.01). On final sRRP pathology, the rates of grade group ≥ 4 (51% vs. 36%, *p* = 0.2), pT3 (69% vs. 75%, *p* = 0.6), positive nodes (30% vs. 18%, *p* = 0.2) and positive margins (33% vs. 39%, *p* = 0.5) were similar for RT versus FT, respectively. The 3‐year biochemical recurrence‐free survival was 86% for RT versus 94% for FT (*p* = 0.6).

**Conclusion**

sRRP for recurrent PCa after FT is associated with lower complications and higher urinary continence and potency rates than patients who received primary RT.

## 1 INTRODUCTION

Aproximately one in four men diagnosed with localized prostate cancer (PCa) opt for nonsurgical primary treatment,^1^ with radiation therapy (RT) being a well‐established option.^2^ Focal therapy (FT) has emerged as an alternative treatment for select patients who desire greater preservation of functional outcomes while achieving satisfactory cancer control.^3^, ^4^ Nevertheless, a proportion of patients undergoing primary ablation or radiation will develop recurrence.^5^, ^6^ Given the absence of standardized strategies to date, the challenge lies in determining the optimal management for these patients.

The complexity of patient decision‐making is further intensified when faced with recurrence. While initial nonsurgical treatments emphasize quality of life preservation, recurrence often necessitates consideration of more aggressive interventions. Options with curative intent for salvage treatment for local failure comprise radical prostatectomy (RP), RT and ablation therapy, with or without androgen deprivation therapy (ADT).^7^, ^8^ Open salvage RP provides high long‐term cancer control rates; however, it has historically been linked to high perioperative complication rates, attributed to the procedure's complexity and the toxicity of prior treatments.^9^ With the advent of robotic approach, recent studies have demonstrated improved outcomes following salvage robotic RP (sRRP).^10^ Hence, current guidelines recommend salvage RP for patients with nonmetastatic recurrent PCa and over 5 years of life expectancy.^11^, ^12^, ^13^

There is limited high‐quality data on the effect of primary treatment modality (i.e., RT vs. FT) on the functional and oncological outcomes of sRRP. To better inform clinicians and patients, it is essential to weigh the aggressiveness of the approach against the acceptable compromise in functional outcomes to achieve oncological control. This study aims to assess perioperative and medium‐term outcomes in patients who underwent sRRP following local recurrence post‐FT or RT.

## 2 MATERIALS AND METHODS

### 2.1 Study population

The records of consecutive patients who underwent sRRP following primary local therapy for PCa (RT or FT) between January 2010 and December 2022 were reviewed from an institutional review board (IRB)‐approved PCa database (IRB no. HS‐012030). Patients with concomitant procedures, including fistula repair and urethroplasty, were excluded.

### 2.2 Pre‐operative assessment

All patients had biopsy‐proven local recurrent PCa without evidence of metastatic disease assessed by the standard of care at the time of recurrence.^13^ Patients were selected for sRRP at the discretion of a multidisciplinary team and provided consent for the operation.

### 2.3 Surgical intervention and follow‐up

Pre‐operatively, the patients received prophylactic anticoagulation and antibiotics. Surgeries were performed with the patients on trendelenburg position, with a 12–15 mmHg pneumoperitoneum, using DaVinci Si or Xi robotic systems via a transperitoneal multiport approach. Six high‐volume urologists, all from the same institution, performed the surgeries. The operations were carried out through the Retzius space followed by the opening of the bladder neck in an antegrade fashion as described elsewhere.^14^ The decision to perform a nerve‐sparing procedure and its extent was made intraoperatively at the surgeon's discretion. The posterior reconstruction stitch was routinely performed, and the bladder‐urethral anastomosis was accomplished with a double‐armed 3–0 V‐Lock continuous suturing. An 18Fr urethral Foley and a 19Fr pelvic drain were routinely placed. An extended bilateral pelvic lymph node dissection was performed on all patients, involving the removal of the obturator, internal iliac, external iliac, common iliac, and the Cloquet lymph nodes.

Postoperatively, the patients followed an enhanced recovery after surgery (ERAS) pathway, which included early ambulation, clear liquid diet on post‐op day 0, and ERAS diet as tolerated, typically on post‐op day 1. A multimodal pain regimen with acetaminophen, gabapentin, and local anaesthetics were used intraoperatively with minimal opioid usage.^15^ The pelvic drain was routinely removed prior to discharge. Prophylactic anticoagulation was maintained for up to 28 days postoperatively. A cystogram was obtained 10–14 days following surgery, if deemed necessary by the surgeon. The catheter was removed per the surgeon's decision and/or if no contrast extravasation was seen in the cystogram. Follow‐up evaluations were conducted according to established PCa guidelines,^11^, ^12^, ^13^ which typically included prostate‐specific antigen (PSA), validated questionnaires, and clinical assessment every 3 months during the first year, every 6 months from the second to the fifth year, and annually thereafter.

The pathology specimens were evaluated by uropathologists according to the International Society of Urological Pathology (ISUP) standards. Grade group was assigned unless the pathologist was unable to determine it due to treatment effects.^16^

### 2.4 Study endpoints and definitions

The primary endpoint was perioperative outcomes, including pathological findings and 90‐day complications. Complications were recorded using the modified Clavien–Dindo grading classification and high grade was defined as grade ≥ 3.^17^

Secondary endpoints included postoperative urinary continence, potency, and biochemical recurrence in FT versus RT groups. Erectile function was assessed by the International Index of Erectile Function (IIEF) questionnaire. Postoperative potency and continence were defined as an IIEF‐5 score of ≥18 and no pad usage, respectively, as previously described.^3^, ^4^ These parameters were assessed in a subgroup of patients who were potent and continent before surgery. Operative time was recorded from the initial incision to the closure of the skin. Biochemical recurrence (BCR) following sRRP was defined as PSA > 0.2 ng/mL.

### 2.5 Statistical analysis

Demographic and clinical features were summarized using the median and interquartile range (IQR) for continuous as well as frequency count and percentage for categorical variables. Associations between clinicopathological characteristics and outcomes were assessed by univariate models, using Fisher's Exact test and Wilcoxon rank sum test for categorical and continuous variables, respectively. Survival from artificial urethral sphincter (AUS) placement and BCR were estimated using the Kaplan–Meier (KM) method, respectively. Logistic regression models were applied to assess predictors for urinary incontinence and potency at 1 year following surgery and overall 90‐day complications. Cox regression analyses were performed to assess variables for BCR recurrence. Statistical software JMP PRO®, version 16 (SAS Institute Inc., NC, USA) was applied to all the analyses in this study. All p‐values reported were two‐sided and *p* < 0.05 was considered statistically significant.

## 3 RESULTS

### 3.1 Baseline clinicopathologic characteristics

A total of 112 patients with a median (IQR) age of 68 (63–74) years were included. Of these, 84 and 28 had received RT and partial gland FT as their primary treatment, respectively. The RT group included external beam RT (*n* = 55), brachytherapy (*n* = 26) and combined external beam RT and brachytherapy (*n* = 3). The FT group included cryoablation (*n* = 15), high‐intensity focused ultrasound (HIFU) (*n* = 11), both HIFU and cryoablation (*n* = 1) and focal laser ablation (*n* = 1).

Baseline clinical features of the patients are shown in Table 1. The time interval between primary treatment and sRRP was significantly longer in the RT versus FT groups (median 83 vs. 38 months, respectively, *p* < 0.001). In addition, pre‐sRRP biopsy showed a significantly higher rate of high‐grade (grade group ≥4) PCa in RT compared with FT (47% vs. 21%, *p* = 0.02).

**TABLE 1:** Baseline features of patients stratified by primary local treatment.

Variable	Total (*n* = 112)	RT (*n* = 84)	FT (*n* = 28)	*p*
Age, median (IQR), year	68 (63–74)	69 (62–74)	67 (63–73)	0.6
Primary treatment: sRRP interval, month	67 (37–108)	83 (47–120)	38 (22–93)	**<0.001**
Pre‐sRRP PSA	5.4 (3.4–8.6)	5.0 (2.9–8.0)	7.3 (4.0–9.6)	0.09
CCI	0 (0–1)	0 (0–1)	0 (0–1)	0.4
History of ADT	23 (21)	18 (21)	5 (18)	0.8
No. ISUP grade group grade group (%)				
1	12 (11)	10 (13)	2 (7)	0.06
2	34 (32)	20 (26)	14 (50)	
3	17 (16)	11 (14)	6 (22)	
4	20 (19)	16 (21)	4 (14)	
5	22 (21)	20 (26)	2 (7)	
≥4	42 (40)	36 (47)	6 (21)	**0.02**

Abbreviations: CCI, Charlson comorbidity index; FT, focal therapy; IQR, interquartile range; ISUP, International Society of Urological Pathology; PSA, prostate specific antigen; RT, radiotherapy; sRRP, salvage robotic radical prostatectomy.

### 3.2 Perioperative and pathological outcomes

All operations were accomplished robotically with no need for open conversion. There was one rectal injury in the RT group, which was repaired intraoperatively. Operative time, estimated blood loss, nerve‐sparing status and length of hospital stay were similar between the two groups (Table 2). Nevertheless, the postoperative catheterization time was longer in RT versus FT groups (median 14 vs. 11 days, respectively; *p* = 0.048).

**TABLE 2:** Perioperative outcomes and pathologic findings stratified by primary local treatment.

Variable	Total (*n* = 112)	RT (*n* = 84)	FT (*n* = 28)	*p*
Operative time (min), median (IQR)	240 (210–270)	240 (210–268)	240 (211–278)	0.2
Blood loss (mL), median (IQR)	100 (100–188)	100 (75–150)	125 (100–200)	0.18
Nerve sparing, *n* (%)				0.7
No	63 (56)	48 (57)	15 (54)	
Unilateral	88 (7)	5 (6)	3 (11)	
Bilateral	41 (37)	31 (37)	10 (36)	
Hospital stay (day), median (IQR)	2 (1–2)	2 (1–3)	2 (1–2)	0.4
Catheterization time (day), median (IQR)	13 (9–18)	14 (9–20)	11 (8–14)	**0.048**
Pathology ISUP grade group, *n* (%)				0.2
1	1 (1)	1 (1.2)	0 (0)	
2	26 (23)	16 (19)	10 (36)	
3	28 (25)	20 (24)	8 (29)	
4	19 (17)	14 (17)	5 (18)	
5	30 (27)	25 (30)	5 (18)	
NA (treatment effect)	8 (7)	8 (9.5)	0 (0)	
Pathology stage, *n* (%)				0.4
T2	3 (3)	2 (2)	1 (4)	
T2a	12 (11)	9 (11)	3 (11)	
T2b	0 (0)	0 (0)	0 (0)	
T2c	17 (15)	14 (17)	3 (11)	
T3a	34 (30)	21 (25)	13 (46)	
T3b	45 (40)	37 (44)	8 (29)	
T4	1 (1)	1 (1)	0 (0)	
No. lymph nodes removed, median (IQR)	17 (10–25)	16 (9–25)	18 (12–25)	0.5
No. patients with positive lymph node (%)	30 (27)	25 (30)	5 (18)	0.2
Margin status, *n* (%)				0.5
Negative	73 (65)	56 (67)	17 (61)	
Focal positive (<3 mm)	13 (12)	8 (9)	5 (18)	
Diffuse positive (≥3 mm)	26 (23)	20 (24)	6 (21)	
90‐day complications, *n* (%)	31 (28)	28 (33)	3 (11)	
Low‐grade	22 (20)	19 (23)	3 (11)	**0.03**
High‐grade	9 (8)	9 (11)	0 (0)	

FT, focal therapy; IQR, interquartile range; ISUP, International Society of Urological Pathology; NA, not applicable; RT, radiotherapy.

Nonorgan‐confined disease (pT ≥ 3) was detected in 80 (71%) cases. There was no statistically significant difference between the two groups in terms of total number of lymph nodes removed, positive nodes, pathological stage and margin status (Table 2). The rates of grade group ≥4 (51% vs. 36%, *p* = 0.2), pT3 (69% vs. 75%, *p* = 0.6), positive lymph nodes (30% vs. 18%, *p* = 0.2) and positive margins (33% vs. 39%, *p* = 0.5) were statistically similar for RT versus FT, respectively.

The overall 90‐day complication rate was 28%, including 8% high‐grade complications. The details of these complications are presented in Table 3. The overall 90‐day complications were significantly higher in RT group compared with the FT group (33% vs. 11%, *p* = 0.03). On multivariable analysis, history of RT (odds ratio [OR] 5.09, 95% confidence interval [CI] 1.34–19.37, *p* = 0.02) and prolonged operative time (OR 1.10 per 10 min, 95% CI 1.02–1.18, *p* = 0.02) were associated with a higher rate of 90‐day complications.

**TABLE 3:** 90‐day complications stratified by grade and primary local treatment.

Grade (*n*)^a^	RT (*n* = 28)	FT (*n* = 3)
Grade I–II		
Urine leak	9	
Haematuria	6	
Acute urinary retention	3	
Deep vein thrombosis	2	
Urinary tract infection	1	1
Low haemoglobin requiring transfusion	1	1
Surgical site infection	1	1
Grade III		
Bladder neck contracture	4	
Lymphocele	1	
Rectal injury	1	
Incisional hernia	1	

Abbreviations: FT, focal therapy; RT, radiotherapy.

^a^ Highest grade per patient is reported.

### 3.3 Functional and oncological outcomes

**FIGURE 1:**
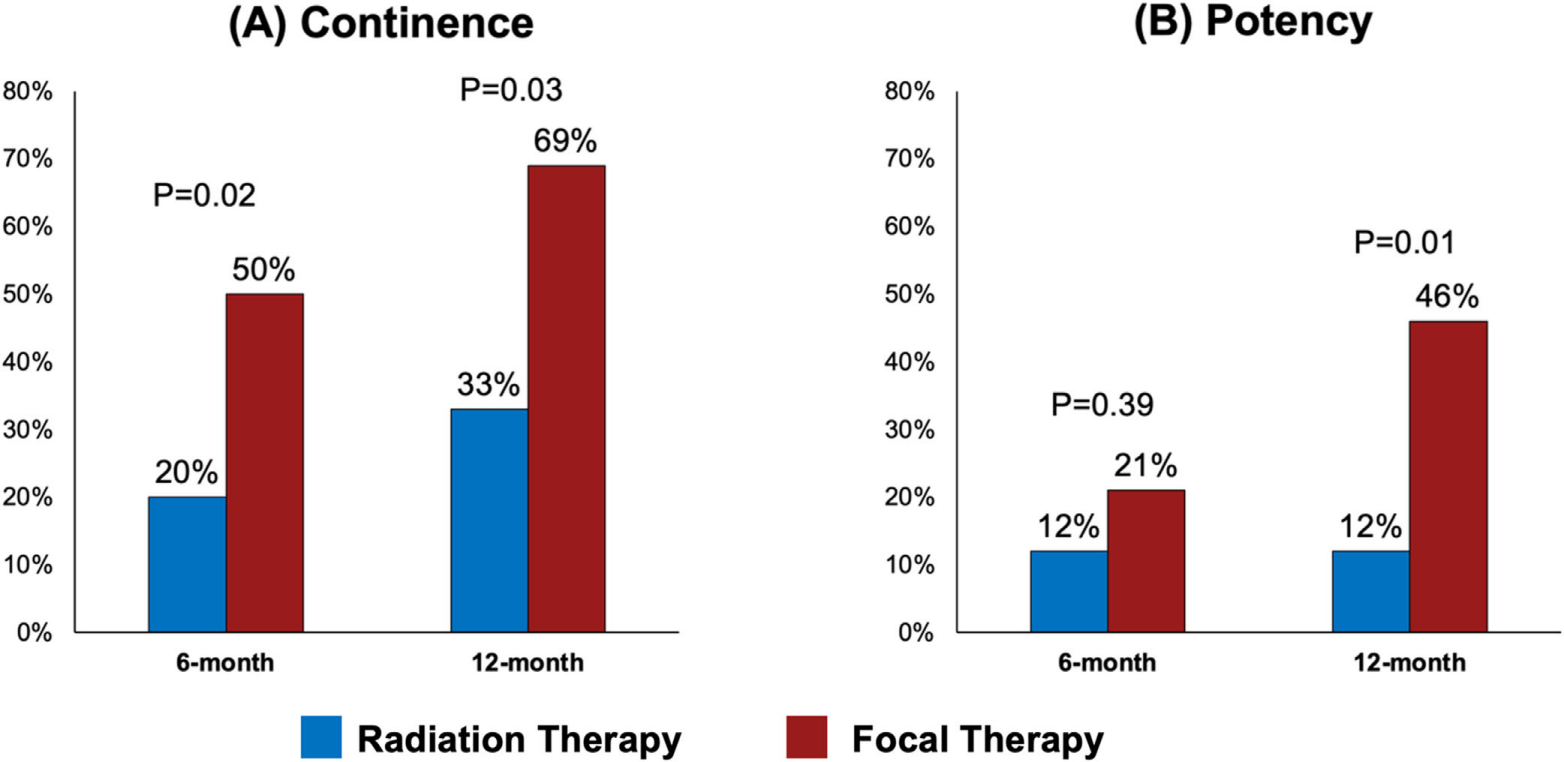
Functional outcomes of salvage robotic radical prostatectomy after radiation therapy versus focal therapy.

The overall continence rates at 6‐ and 12 months following sRRP were 27% and 44%, respectively. These rates were significantly higher in the FT group compared with the RT group (6 months: 50% vs. 20%, *p* = 0.02 and 12 months: 69% vs. 33%, *p* = 0.03) (Figure 1A). On multivariable analysis, a history of RT was associated with a significantly higher rate of urinary incontinence at 12 months following surgery (OR 4.14, 95% CI 1.15–14.90, *p* = 0.03). On KM analysis, the 2‐year AUS placement‐free survival rate was 55% in the RT group versus 94% in the FT group (*p* = 0.002).

The overall potency rates at 6‐ and 12 months following sRRP were 14% and 19%, respectively. Potency at 12 months was better preserved in the FT group compared with the RT group (46% vs. 12%, *p* = 0.01) (Figure 1B). On multivariable analysis, history of RT was associated with a significantly higher rate of 12‐month impotency (OR 5.49, 95% CI 1.34–22.53, *p* = 0.02).

**FIGURE 2:**
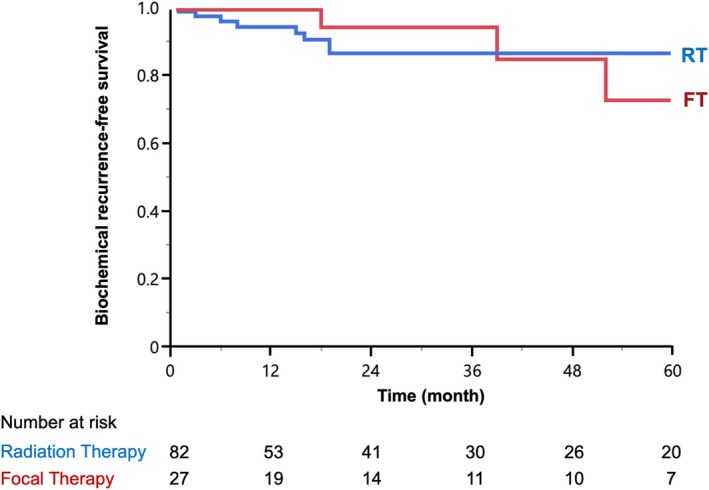
Kaplan–Meier analysis, demonstrating the estimated probability of biochemical recurrence‐free survival for salvage robotic radical prostatectomy after radiation therapy (RT) and focal therapy (FT).

The median (IQR) follow‐up time was 28 (7–64) months, which was similar between the two groups (RT = 30 vs. FT = 26 months; *p* = 0.9). On KM analysis, the 3‐year BCR‐free survival rate was 86% in the RT group versus 94% in the FT group (*p* = 0.6) (Figure 2). Multivariable analysis demonstrated that there was no difference in the BCR between FT and RT groups (hazard risk [HR] 0.94, 95% CI 0.26–3.43, *p* = 0.9). However, grade group of the prostatectomy specimen was an independent predictor for BCR (HR 1.94 for each grade increase, 95% CI 1.17–3.5, *p* = 0.02).

## 4 DISCUSSION

This study represents one of the largest single‐centre investigations of sRRP. Our findings indicate that sRRP following RT or FT is feasible, although there is a notable incidence of perioperative complications, particularly among those with a history of RT. Additionally, compared with FT, a history of RT has a negative impact on perioperative and functional outcomes following sRRP.

Our study demonstrated that most patients with recurrent disease following local treatment for PCa present with advanced stages on final whole‐mount pathology (T3 ≈ 70% and positive nodes ≈ 25%). These findings align with existing literature, which indicates that up to 75% and 60% of men with recurrent PCa have ≥pT3 and pN+ disease, respectively.^18^ Additionally, contrary to some studies showing adverse pathological features after FT versus RT,^19^, ^20^ our study demonstrated comparable pathological characteristics (i.e., stage, ISUP grade and nodal involvement) between these two groups.

The high‐risk pathological features among recurrent PCa cases might lead to BCR. The average 5‐year BCR‐free survival rate following salvage RP has been reported to be as high as 50%.^18^ As expected, this rate is significantly lower than that of treatment‐naïve cases.^21^, ^22^ Despite BCR following sRRP, studies have demonstrated acceptable oncological outcomes with a low incidence of metastasis and cancer‐related mortality.^10^ Nevertheless, oncological outcomes in recurrent cases following FT versus RT are comparable. In a study of sRRP post‐primary RT versus FT, BCR‐free survival rates were similar (59% vs. 56%, respectively; *p* = 0.76).^19^ The current study demonstrated similar findings with a 3‐year BCR‐free survival rate of 86% in the RT group and 94% in the FT group. The better oncological outcomes observed in our study compared with prior reports might be attributed to the high level of experience among urologists involved in the management of PCa at our referral centre, which is also reflected in a lower positive surgical margin rate. It is important to note that the outcomes might have been influenced by the initial patient selection, as patients receiving FT may have had more favourable disease characteristics compared with those receiving RT.

One of the major challenges of sRRP is the intense distortion of tissue planes following initial treatment, which makes tissue dissection difficult. This can potentially increase the perioperative complications in the salvage setting. In a recent systematic review of salvage RP studies, the rate of perioperative high‐grade complications ranged between 0% and 64%.^18^ With the widespread use of minimally‐invasive approaches, particularly robotic and improvement in delivering radiation therapy, the complication rates have decreased significantly in recent years.^10^ Similar trend has been reported for rectal injuries, with an 0.8% rate in the contemporary series compared with 3.7% in older studies.^10^ In the present series, the rate of 90‐day high‐grade complications was 8%, and only one patient experienced rectal injury. Interestingly, albeit not unexpectedly, history of RT was associated with an increased rate of 90‐day complications (OR 5.09).

Another concern when selecting a patient for salvage RP is the potential compromise of functional outcomes. Nerve sparing, which is a major factor contributing to better functional outcomes, might be challenging in these cases due to local treatment‐related reactions and invasion of the neurovascular bundle by PCa. Nerve‐sparing procedures have been reported in up to 36% of patients undergoing salvage RP.^10^ Similar rate was detected in the current series (37% bilateral and 7% unilateral nerve sparing). Assessing erectile function in these patients is challenging given the heterogeneity of contributing factors, including initial treatment modalities, interval between initial treatment and salvage surgery, and history of ADT. In our series, among those without erectile dysfunction before sRRP, potency at 12 months was better preserved in the FT group compared with the RT group, and history of RT was an independent factor associated with impotency after surgery. The continence rate following salvage cases is lower than that after primary RP. A recent systematic review reported full continence (zero pad) rate of 40.5% at 12 months following salvage RP.^10^ Nevertheless, better continence rates have been reported in post‐FT series.^23^, ^24^, ^25^ In a comparative study, full continence rate at 12 months was 77.3% versus 39.2% in salvage RP following FT versus RT (*p* = 0.002).^19^ Similar findings were observed in our series, where a history of RT was identified as an independent factor affecting urinary incontinence at 12 months following surgery. The notably lower rates of potency and continence in cases of radio‐recurrent PCa are important and should be discussed with patients during preoperative counselling for sRRP. These findings may be attributed to the greater likelihood of preserving the functional integrity of pelvic structures after FT, in contrast to the more extensive periurethral tissue damage and vascular compromise following RT, as indicated by the higher rates of postoperative urine leakage and bladder neck contracture.

This study has limitations. Firstly, the retrospective nature of the study resulted in missing values, particularly for functional outcomes. Nonetheless, the data were prospectively maintained, ensuring the quality of the data included. Secondly, the findings might have been affected by selection bias due to the small group of patients treated in a single tertiary referral centre which were selected for treatment at the multidisciplinary team's discretion. Yet the single‐centre focus ensures consistency in surgical techniques and patient management, with all surgeries performed by highly skilled urologists with considerable experience in robotic surgery. Lastly, long‐term oncological outcomes were also unavailable.

In conclusion, the primary local treatment modality affects the outcomes of sRRP for recurrent PCa. Those with initial RT, compared with FT, have higher rates of perioperative and higher‐grade complications. In addition, primary FT is associated with higher urinary continence and potency rates. Most patients presented with locally advanced disease on final whole‐mount pathology; however, with acceptable medium‐term oncological outcomes, which are similar between these two groups.

## AUTHOR CONTRIBUTIONS

*Conceptualization*: A.L.A. and M.A. *Methodology*: A.L.A, A.G., and L.S.R. *Validation*: A.G. and L.S.R. *Formal analysis*: M.K. *Investigation*: A.G., L.S.R., L.G.M., G.E.C, S.K., M.A., S.S., H.J., H.D., R.S., M.M.D., I.S.G., M.A., and A.L.A. *Data curation*: A.G., L.S.R, and M.K. *Writing—original draft preparation*: A.G and L.S.R. *Writing—review and editing*: L.G.M., G.E.C., M.A., S.S., H.J., H.D., R.S., M.M.D., I.S.G., M.A., and A.L.A. *Visualization*: A.G., L.S.R., and M.K.; *Supervision*: A.L.A.

## CONFLICT OF INTEREST STATEMENT

Andre Abreu: proctor and speaker for Sonablate, speaker for EDAP and is proctor and has research grant with Koelis. Inderbir Gill: OneLine Health, EditorAIpro. Hossein Jadvar: on the advisory boards of Pharmalogic and Lantheus; on the speaker's bureau for Lantheus and Blue Earth Diagnostics; and a consultant to Bayer and Siemens. The rest of the authors declare no conflict of interest.
